# Effect of copper ions on the associations of Azospirillum bacteria with wheat seedlings (Triticum aestivum L.)

**DOI:** 10.18699/VJGB-22-58

**Published:** 2022-08

**Authors:** A.Yu Muratova, E.V. Lyubun, S.N. Golubev, O.V. Turkovskaya

**Affiliations:** Institute of Biochemistry and Physiology of Plants and Microorganisms – Subdivision of the Saratov Federal Scientific Centre of the Russian Academy of Sciences, Saratov, Russia; Institute of Biochemistry and Physiology of Plants and Microorganisms – Subdivision of the Saratov Federal Scientific Centre of the Russian Academy of Sciences, Saratov, Russia; Institute of Biochemistry and Physiology of Plants and Microorganisms – Subdivision of the Saratov Federal Scientific Centre of the Russian Academy of Sciences, Saratov, Russia; Institute of Biochemistry and Physiology of Plants and Microorganisms – Subdivision of the Saratov Federal Scientific Centre of the Russian Academy of Sciences, Saratov, Russia

**Keywords:** Azospirillum, Triticum aestivum, copper, seedlings, photosynthetic pigments, peroxidase, laccase, tyrosinase, Azospirillum, Triticum aestivum, медь, проростки, фотосинтетические пигменты, пероксидаза, лакказа, тирозиназа

## Abstract

The physiological and biochemical activity of plant–microbial associations enables them to determine the mobility, bioavailability, and accumulation of heavy metals in plant tissues. These abilities are the basis for the use of plants and their associated microorganisms in the development of approaches that ensure both the prevention of the ingress of toxic metals into food crops and the extraction of pollutants from polluted soils by using phytoremediation technologies. Whether plant–microbial complexes are used successfully depends on the knowledge of how specific organisms interact with heavy metals. We evaluated the effect of copper ions on common wheat (Triticum aestivum L.) inoculated with three plant-growth-promoting rhizobacteria (PGPR) of the genus Azospirillum. We analyzed the growth variables of 14-day-old wheat seedlings, the content of photosynthesis pigments, the activity of plant oxidoreductases, and the accumulation of copper by plant tissues. All strains more or less compensated for copper toxicity to seedling development and increased metal accumulation in roots and shoots. Copper affected the photosynthetic apparatus of the inoculated plants, primarily by decreasing the content of chlorophyll b. An analysis of the activity of plant oxidoreductases (peroxidases and phenoloxidases), which are involved in the physiological responses of plants to pollutant stress, showed strain-specific dependence and a significant effect of copper on the inoculated plants. Overall, the obtained results clearly show that the effect of Azospirillum on the physiological and biochemical status of wheat is diverse. The compensatory effect of bacteria on copper toxicity and the simultaneous increase in metal accumulation in plant tissues can be considered as mutually exclusive crop-production aspects associated with the growing of food plants in heavy-metal-polluted areas.

## Abstract (ru)

Растительно-микробные ассоциации в результате своей физиолого-биохимической активности способны определять подвижность, биодоступность и накопление в растительных тканях тяжелых металлов. Указанные способности являются основой для использования растений и ассоциированных с ними микроорганизмов в разработке подходов, обеспечивающих как предотвращение попадания токсичных металлов в пищевые культуры, так и извлечение поллютантов из загрязненных земель с помощью технологий фиторемедиации. Успешное применение растительно-микробных комплексов в той или иной области зависит от изученности механизмов взаимодействий в системе конкретных организмов с тяжелыми металлами. Целью представленных исследований была оценка влияния ионов меди на эффекты бактеризации растений пшеницы мягкой (Triticum aestivum L.) тремя штаммами Azospirillum, обладающими свойствами стимуляции роста растений (PGPR). В ходе эксперимента анализировали ростовые параметры 14-суточных проростков пшеницы, содержание пигментов фотосинтеза, активность растительных оксидоредуктаз и аккумуляцию металла растительными тканями. Все штаммы в той или иной степени компенсировали фитотоксическое воздействие меди на развитие проростков и увеличивали ее аккумуляцию в корнях и побегах. Показано отчетливое усиление воздействия меди на фотосинтетический аппарат бактеризованных растений, выражающееся в изменении содержания основных пигментов, в первую очередь уменьшении хлорофилла b. Анализ активности растительных оксидоредуктаз (пероксидаз и фенолоксидаз) как участников физиологических ответов растений на стрессовые воздействия выявил их штаммоспецифичный характер и существенное влияние меди на бактеризованные растения. В целом полученные результаты показали отчетливое разноплановое влияние исследованных штаммов азоспирилл на физиолого- биохимический статус растений пшеницы. Выявленный компенсаторный эффект бактерий на фитотоксическое воздействие меди и одновременно повышение ее накопления в растительных тканях могут рассматриваться как взаимоисключающие аспекты растениеводства, связанные с выращиванием пищевых растений на загряз- ненных тяжелыми металлами площадях.

## Introduction

Soil pollution by heavy metals is a serious environmental problem. The accumulation of heavy metals in ecosystems leads to their increased uptake by plants and migration along food chains up to humans (Larionov M.V., Larionov N.V., 2010). Plant–microbial complexes are most important for the transformation, translocation, and accumulation of heavy metals in nature. The physiological and biochemical activity of microorganisms and plants enable them to transform heavy metal compounds and determine metal mobility, bioavailability and accumulation (Nadeem et al., 2015). These abilities are the basis for the use of plant–microbial associations in the development of approaches to prevent the input of toxic metals into food crops, on the one hand, and in the development of technologies for cleaning agricultural landscapes from pollutants (phytoremediation), on the other hand. Whether plant–microbial complexes are used successfully depends on the knowledge of how specific organisms interact with heavy metals.

The vital activity of Azospirillum bacteria, typical members of the associative microflora of plants, is closely linked to the root system of plants, mainly that of cereals (Reis et al., 2015). Azospirilla are facultative diazotrophs that can fix atmospheric nitrogen under microaerophilic conditions and produce phytohormones (auxins, gibberellins, and cytokinins) and other phytoactive substances. This makes them prominent plant-growth-promoting rhizobacteria (PGPR) (Bashan, De- Bashan, 2010; Fukami et al., 2018). Azospirilla use different strategies to colonize plant roots, which enables differentiation between epiphytic strains (those able to colonize only the root surface) and endophytic strains (those able to penetrate into the root interior) (Rothballer et al., 2003). By interacting with plants, azospirilla promote their growth and reduce environmental stress through various mechanisms, including increased mobilization and absorption of minerals (Bashan, De-Bashan, 2010). A typical associative plant for azospirilla is wheat. Inoculation with Azospirillum is beneficial to agriculturally important crops, including wheat (Teixeira Filho et al., 2017; Galindo et al., 2019; Boleta et al., 2020).

Members of the species A. brasilense are resistant to a number of toxic metals (Co, Cu, Zn, and Cd). Endophytic and epiphytic azospirilla differ markedly in their resistance to metals (Kamnev et al., 2005, 2007).

Copper is a very important trace element involved in various plant physiological processes, such as electron transport during photosynthesis, mitochondrial respiration, response to oxidative stress, and hormonal signaling. As a cofactor, this metal is part of many plant enzymes and proteins, such as superoxide dismutase, cytochrome c oxidase, amino oxidase, laccase, tyrosinase, polyphenol oxidase, and plastocyanin (Yruela, 2005; Pichhode, Nikhil, 2015). However, high concentrations of copper are phytotoxic and cause various kinds of damage to plants, including wheat (Quartacci et al., 2000; Michaud et al., 2007; Dang et al., 2009). Inoculation with A. brasilense increases wheat resistance to stress caused by the presence of Cu2+ ions (El-Samad, 2017). Yet, inoculation with strains that use different strategies to interact with plants may differ in its effect, which requires additional study. Previous studies (Kamnev et al., 2007) showed that epiphytic and endophytic A. brasilense strains differ in the mechanisms of metal resistance, which is linked to the accumulation of poly- 3-hydroxybutyrate as a factor contributing to survival under adverse conditions. On the basis of those results, we assumed that under heavy metal stress, such strains may differ in their interaction with plants

We examined the effect of copper ions on soft wheat (Triticum aestivum L.) inoculated with different plant-growthpromoting strains of A. brasilense.

## Materials and methods

Azospirillum brasilense Sp7 (IBPPM 150), A. brasilense Cd (IBPPM 288), and A. baldaniorum Sp245 [IBPPM 219, formerly A. brasilense Sp245 and reclassified by dos Santos Ferreira et al. (2020)], from the IBPPM RAS Collection of Rhizosphere Microorganisms (http://collection.ibppm.ru) were used in this study.

Bacteria were grown in a liquid or on an agarized (1.5 %) medium composed as follows (g/L): K2HPO4 – 0.1; KH2PO4 – 0.4; MgSO4·7H2O – 0.2; NaCl – 0.1; CaCl2 – 0.02; FeSO4 · 7H2O – 2.0; NTA-3Na – 5.6; NaMoO4·2H2O – 0.002; sodium malate, 3.8; NH4Cl – 1.0; pH 7.0. Copper was used as a copper sulfate at 0.5 mmol/L, which, according to preliminary studies, is the minimal concentration inhibiting bacterial growth.

Seeds of soft spring wheat (Triticum aestivum L. cv. Saratovskaya 29) were obtained from the Federal State Budgetary Scientific Organization “Federal Center of Agriculture Research of the South-East Region”. After being calibrated, the seeds were washed with a detergent for 10 min with shaking to remove hydrophobic contaminants and were sterilized with 70 % (vol./vol.) ethanol for 3 min, then with diacide (1:1000; 666 mg/L cetylpyridine chloride and 333 mg/L ethanol mercury chloride) for 5 min, then with a mixture of rifampicin (4 μg/mL) and amphotericin B (20 μg/mL) at room temperature for 24 h with shaking (120 rpm), and finally with diacide (1:1000) for 2.5 min. After each stage, the seeds were repeatedly washed with sterile distilled water. The sterilized seeds were placed one by one in sterile biological tubes (20 × 300 mm) containing 15 cm3 glass beads with a diameter of 2 mm (SiLibeads, Sigmund Lindner,Warmensteinach, Germany) and 6 mL of Hoagland’s solution for plant growth (Hoagland, Arnon, 1950). CuSO4 · 5H2O was added to the growth medium to achieve a Cu2+ ion concentration of 0.5 mmol/L. The control medium was copperfree. The tubes with experimental plants were inoculated with a microbial suspension. 

For inoculation, bacteria were grown in a liquid malate medium for 18 h, after which they were centrifuged (11 000 g, 5 min), washed twice, and resuspended in a sterile medium. Each tube with a 3-day-old seedling was inoculated with 30 μL of bacterial suspension to an inoculant concentration of 107 cells per mL in the plant growth solution. Noninoculated plants were used as the control. The plants were grown under controlled conditions at 24 °C for 14 days with a 13/11 h day/night illumination period. Lighting was provided by Fluora fluorescent lamps (Osram, Munich, Germany).

At the end of plant growth, we analyzed the growth variables of 14-day-old seedlings, the content of photosynthesis pigments, the activity of plant oxidoreductases, and the accumulation of copper in plant tissues. The morphological variables (root and shoot length) were measured with a calibrated stainless ruler. The roots and shoots were then dried to a constant weight.

Biochemical analysis of seedlings included the measurement of the content of photosynthetic pigments and the examination of the enzyme activity of roots and shoots. The content of chlorophylls a and b (Chl a and Chl b) and carotenoids was determined spectrophotometrically in ethanol leaf extracts, as described earlier (Lyubun et al., 2020).

For determining the activity of plant oxidoreductases (peroxidases, laccases, and tyrosinases), shoots and roots (0.2–0.3 g) were ground in a mortar with quartz sand and were resuspended in 2 mL of 0.2 M Na/K phosphate buffer (pH 6.0). The homogenate was centrifuged at 5000 g for 10 min, the sediment was additionally washed with a phosphate buffer and was recentrifuged. Enzyme activity and protein content were determined in the resultant combined supernatants by using an Evolution 60 spectrometer (Thermo Scientific, USA). The protein content was determined by the Bradford method (Bradford, 1976).

Peroxidase activity (EC 1.11.1.7) was measured by using 23 μM of 2,7-diaminofluorene (DAP) in 0.05 M Na/K-phosphate buffer (pH 6.0) at 600 nm (Criquet et al., 2000); 1 mM 2,2-azino-bis(3-ethylbenzothiazoline-6-sulfonate) ammonium (ABTS) in 0.05 M Na-tartrate buffer (pH 3.5) at 436 nm (Yang et al., 2007); and 0.3 mM o-dianisidine (DAZ) in 0.05 M Na/K-phosphate buffer (pH 6.0) at 460 nm in the presence of 0.5 mM H2O2. Laccase activity (EC 1.10.3.2) was determined by the formation of the oxidation products of 7.5 μM syringaldazine (SGZ) in 0.05 M Na/K-phosphate buffer (pH 6.0) at 525 nm (Leonowicz, 1981) and 23 μM DAP in 0.05 M Na/K-phosphate buffer (pH 6.0) at 600 nm (Criquet et al., 2000). Tyrosinase activity (EC 1.10.3.3) was determined in a 4 mM solution of 3,4-dihydroxyphenyl-L-alanine (DOPA) and 50 mM Tris-HCl (pH 7.5) at 475 nm (Criquet et al., 2000). Enzyme activity was expressed in mmol of oxidized substrate per min per mg of protein.

The total plant content of copper was analyzed with an atomic absorption spectroscopy system equipped with a graphite furnace (Thermo Scientific iCE 3500 Solaar). A 3-mL portion of HNO3 (Suprapur; Merck, Darmstadt, Germany) and 2 mL of H2O2 (30 %; JTBaker Chemical Co., Philipsburg, New Jersey, USA) were added to Teflon containers containing 200 mg of plant material. The samples were then processed in a CEM MARS Xpress microwave digester (Matthews, NC, USA) by using an optimized program. After processing, the volume of the samples was adjusted to 20 mL with ultrapure deionized water and was analyzed for metal content by spectrometry.

All experiments and analyses were carried out in at least three replicates, each replicate using five to eight plants. Means were compared by Student’s t test (p ≤ 0.05). Correlation analysis was conducted by using Spearman rank correlations. Microsoft Excel 2007 (Microsoft Office, USA) and Statistica 13.0 (TIBCO Software Inc. 2017, Statsoft Russia) software were used for statistical analysis.

## Results

Plant growth

Copper was significantly toxic to seedling development, decreasing both length and biomass of roots and shoots. The inhibition of root growth was more pronounced (root length decreased by 58 % and root weight by 13 %), as compared with shoots, whereas shoot length decreased by a mere 8 % and shoot biomass weight was not changed significantly (Table 1).

**Table 1.**
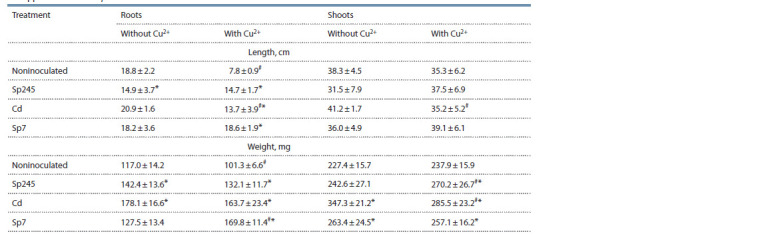
Length and dry weight of cv. Saratovskaya 29 seedlings grown in the presence of copper ions and Azospirillum strains Values represent means (n ≥ 6) ± standard deviation. Here and in the Tables 2–4: * values differ significantly from noninoculated control, p ≤ 0.05; # values differ significantly from copper-free treatment, p ≤ 0.05.

The effect of inoculation on seeding development within 14 days under copper-free conditions depended on the strain used (see Table 1). A. brasilense Sp7 had a significant effect only on shoot biomass, with an increase of 16 %, as compared with the noninoculated control. A. baldaniorum Sp245 significantly reduced root length (by 20 %) and increased root biomass (by 22 %). A. brasilense Cd affected the seedlings the most, significantly increasing root and shoot biomass (by 52 and 53 %, respectively).

The effect of inoculation on root and shoot length and biomass was changed by copper. In seedlings inoculated with strain Cd, root biomass tended to decrease and root length decreased by 34 %. The shoot length and biomass of the seedlings inoculated with strain Cd decreased by 14 and 18 %, respectively. The effect of copper on the root and shoot length of the plants inoculated with strains Sp245 and Sp7 was nonsignificant. Yet, strain Sp245 reduced root biomass but increased shoot biomass (by 11 %); by contrast, strain Sp7 increased root biomass by 33 % and slightly reduced shoot biomass.

With all three strains, inoculation reduced copper toxicity to seedlings, which was most evident as increases in root length (by 1.7–2.4 times) and root biomass (by 30–68 %). The negative effect of copper on root length was fully mitigated only with strain Sp7. Inoculation with all strains not only compensated for the effect of copper on root biomass but also significantly increased it relative to the copper-free noninoculated control.

Content of photosynthesis pigments

In noninoculated seedlings, copper had only a slight effect on the content of and ratio between photosynthesis pigments (Table 2).

**Table 2.**
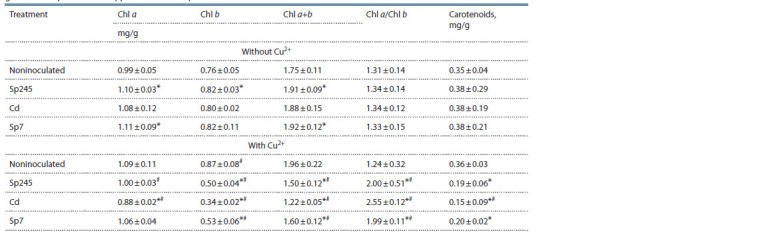
Content of photosynthesis pigments in cv. Saratovskaya 29 seedlings grown in the presence of copper ions and Azospirillum strains Values represent means (n ≥ 6) ± standard deviation

Inoculation of seedlings grown without copper promoted the content of chlorophylls a and b and their total amount by 11, 8, and 9 %, respectively, with strain Sp245, and by 12, 8, and 10 %, respectively, with strain Sp7. The ratio between chlorophylls a and b and the carotenoid content changed slightly in response to treatment with strains Sp245 and Sp7.

With strains Sp245, Cd, and Sp7, the effect of copper was manifested in two ways: (1) significant decreases in the content of the pigments, mostly chlorophyll b (by 43, 58, and 39 %, respectively); in the total content of chlorophylls a and b (by 23, 38, and 18 %, respectively); and in the content of carotenoids (by 47, 58, and 44 %, respectively). (2) Significant increases in the Chl a/Chl b ratio (by 61, 105, and 60 %, respectively).

Accumulation of copper in seedling tissues

The content of copper ions in the tissues of seedlings grown in a copper-free environment varied from 17 to 28 μg/g of dry biomass. The content of copper ions in the seedlings grown in the presence of 0.5 mmol/L of copper is given in the Figure.

**Fig. 1.**
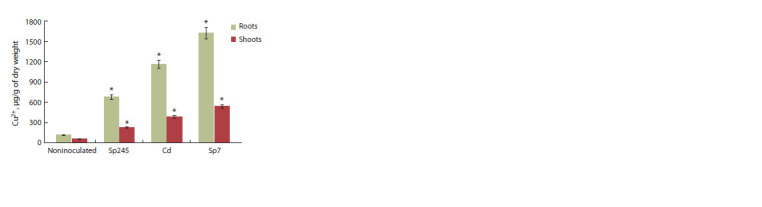
Copper content in dried biomass of cv. Saratovskaya 29 seedlings grown in the presence of copper ions and Azospirillum strains. Values differ significantly from noninoculated control, p ≤ 0.05.

In noninoculated seedlings, the accumulation of copper in the roots and shoots was 116 and 59 μg/g, respectively. These variables were strongly increased by inoculation with Azospirillum. Treatment with strains Sp245, Cd, and Sp7 increased the accumulation of copper in roots by 6, 10, and 14 times and in shoots by 4, 7, and 9 times, respectively

Activity of oxidative enzymes of wheat seedlings

The activity of the total peroxidase of wheat plants was measured by using several substrates, which allowed us to take into account different isoforms of this enzyme. In general, according to our data (Table 3), the activity of peroxidase was significantly higher in roots than in shoots.

**Table 3.**
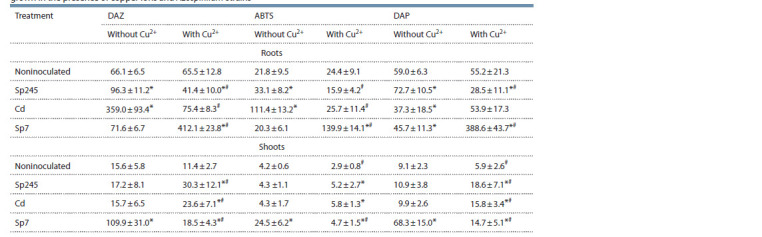
Peroxidase activity (U/mg of protein) in cv. Saratovskaya 29 seedlings grown in the presence of copper ions and Azospirillum strains Test substrates: DAZ, o-dianisidine; ABTS, 2,2-azino-bis(3-ethylbenzothiazoline-6-sulfonate) ammonium; DAP, 2,7-diaminofluorene

The presence of copper in the growth solution at the concentration used did not cause a significant change in root peroxidase activity, but peroxidase activity tended to decrease in shoots.

Inoculation of seedlings with strains Sp245 and Cd increased DAZ and ABTS peroxidase activity by more than 1.5 and 5 times, respectively, in roots but not in shoots. With strain Sp7, the enzymatic response to inoculation was different. In roots the activity of DAZ and ABTS peroxidases remained unchanged, whereas in shoots the activity of DAZ, ABTS, and DAP peroxidases increased by 7.0, 5.8, and 7.5 times, respectively. With strains Cd and Sp7, the activity of DAP peroxidase in roots decreased.

In the presence of copper, the peroxidase activity of the wheat seedlings inoculated with strain Sp7 was sharply increased in roots (by 6.3, 5.7, and 7 times) and was less increased in shoots (by 1.6, 1.6 and 2.5 times) for DAZ, ABTS, and DAP, respectively. In the shoots of seedlings inoculated with strains Cd and Sp245, copper increased peroxidase activity by two to three times.

The measured results for the activity of copper-containing plant phenol oxidases (laccase and tyrosinase) are given in Table 4. In noninoculated seedlings, the addition of copper to the growth solution increased laccase activity in roots (by 1.5 times, with DAP), and decreased it in shoots (by 1.75 times, with SGZ).

**Table 4.**
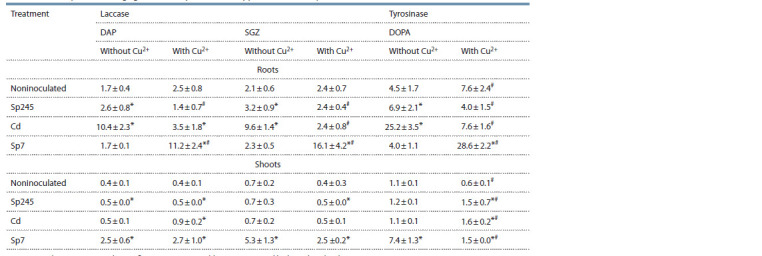
The activity of copper-containing phenol oxidases (laccase and tyrosinase) in cv. Saratovskaya 29 seedlings grown in the presence of copper ions and Azospirillum stra Test substrates: DAP, 2,7-diaminofluorene; SGZ, syringaldazine; DOPA, 3,4-dihydroxyphenyl-L-alanine.

Depending on the strain used, inoculation significantly changed laccase activity. Strain Cd caused the most significant increase in root laccase activity (by 6 times, with DAP), and strain Sp7 promoted shoot laccase activity (by 7.5 times, with SGZ). Copper reduced the effect of inoculation. With strains Sp245 and Cd, the laccase activity of the roots was comparable to that in noninoculated plants (with SGZ). By contrast, strain Sp7 promoted laccase activity by almost 7 times. In the presence of copper, the laccase activity in the shoots of inoculated seedlings varied depending on the strain and test substrate. The activity increased the most (by 1.8 times, with DAP) with strain Cd and decreased (by 2 times, with SGZ) with strain Sp7.

Similar to laccase activity, tyrosinase activity in the presence of copper increased in roots and decreased in shoots (by 1.7 times in either case). Without copper, strains Sp245 and Cd promoted tyrosinase activity by 1.5 and 5.6 times, respectively, in roots but not in shoots, whereas strain Sp7, on the contrary, promoted tyrosinase by 6.7 times in shoots but not in roots. Copper reduced the effect of inoculation with strains Sp245 and Cd on tyrosinase activity in roots (by 1.7 and 3.3 times, respectively) and slightly increased it in shoots. By contrast, in the presence of copper, strain Sp7 promoted tyrosinase activity in roots (7-fold) and decreased it in shoots (5-fold).

## Discussion

Copper is an essential trace element. Excessive copper, however, inhibits plant growth and causes metabolic disorders (Yruela, 2005; Michaud et al., 2007; Wang H. et al., 2011; Pichhode, Nikhil, 2015). This element is widely involved in various physiological processes (photosynthesis, respiration, antioxidant response, hormonal signaling), and a violation of the copper balance can lead to multiple damage to the plant. The mechanism of the toxicity of copper is associated with its ability to bind strongly to oxygen, nitrogen, and sulfur atoms, which, under conditions of excess copper, gives rise to additional bonds and/or substitution of other metals with copper in various biomolecules, including in the active centers of many enzymes (Yruela, 2005; Wang H. et al., 2011). Copper toxicity to plants is manifested as inhibition of growth and signs of chlorosis and is accompanied by oxidative stress. The copper uptake and content in plants depend on several factors, including cultivar differences (Medvedev, Derevyagin, 2017).

In this work, all experiments were conducted with one cultivar of soft spring wheat. This means that the inoculation and copper effects found for cultivar Saratovskaya 29 may not be manifest in other wheat cultivars. Therefore, further studies on different wheat genotypes are needed.

Copper at 0.05 mmol/L affected mainly the wheat seedling roots, which were in direct contact with the toxicant. Wang H. et al. (2011) reported decreases in the length and weight of wheat roots grown hydroponically in the presence of 0.05 mmol/L of copper. Shoot length was also reduced, a trend noted in this study as well. Yet, the copper concentration used had no noticeable effect on the photosynthetic apparatus of wheat. This was concluded from the absence of significant changes in the leaf content of chlorophylls and carotenoids under the effect of the metal (see Table 2). Consequently, photosynthesis was undisturbed by copper toxicity to wheat roots.

The normal physiological concentration of copper in plants ranges from 3 to 30 mg/kg (Wang H. et al., 2011). Wheat is able to take up copper from soil, and roots accumulate larger amounts of copper than does aboveground biomass (Sayyad et al., 2009). Increased metal absorption is an undesirable property of food grains. Liu et al. (2021) examined the genetic mechanisms of metal accumulation by plants by using 246 wheat cultivars and two metals – copper and zinc. They showed that some cultivars are the least prone to the accumulation of toxic elements. The uptake of metals by plants from soil is affected by microbial activity, as well as by numerous organic and inorganic compounds released by roots and present in soil solution (Wang S. et al., 2017). Microbes produce extracellular polymer compounds that can adsorb or chelate metal ions (Yaneva, 2009); as a result, metals are deposited into the medium and are taken up by roots in greater amounts (Wang S. et al., 2017).

Our results show that inoculation of wheat with A. brasilense contributed to copper accumulation in plant tissues. The degree of influence of the inoculants (Sp7 > Cd > Sp245) on this variable is probably related to the differences in the root colonization strategy between bacteria. Strain Sp245 is an endophyte, whereas strain Sp7 is an epiphyte (Rothballer et al., 2003). The available information about strain Cd is contradictory: de Oliveira Pinheiro et al. (2002) failed to observe wheat root penetration by this strain, whereas Caiola et al. (2004) observed Cd cells in the tissues of tomato roots.

Here, A. brasilense inoculation of wheat contributed to increased plant growth, which was manifested mainly as increased length and weight of roots and shoots. Yet, the strains differed in their ability to promote plant growth. The endophytic strain Sp245 inhibited root growth in the absence of copper but compensated for the inhibitory effect of copper in its presence. Strain Cd had the greatest effect on the length and weight of the seedlings grown both in the presence and in the absence of copper.

In turn, copper accumulation in wheat tissues was toxic to plants. All inoculated plants showed a sharp twofold decrease in the content of chlorophyll b and carotenoids (see Table 2), which could not but disturb the photosynthesis apparatus. It is known that high concentrations of copper can suppress photosynthesis, disrupting the architecture of thylakoid membranes, changing the whole ultrastructure of chloroplasts, and inhibiting the accumulation of chlorophyll and the electron transport of both PS I and PS II (Rai et al., 2016). The toxic effect of copper on the photosynthesis apparatus may be associated with inhibition of the activity of biosynthesis enzymes and with the displacement of Mg2+ from the chlorophyll molecule (Prasad M.N.V., Strzalka, 1999; Rai et al., 2016). When the content of the main photosynthetic pigment chlorophyll a decreases (which is what was observed when strain Cd was used for inoculation), the auxiliary chlorophyll b converts to chlorophyll a. As a result, the concentration of chlorophyll b decreases to a greater extent than does the concentration of chlorophyll a, and ultimately, the chlorophyll a/b ratio increases (Breckle, 1991; Prasad D.D.K., Prasad A.R.K., 1987). With strains Sp245 and Sp7, which did not affect the chlorophyll a content in the inoculated leaves in the presence of copper, the chlorophyll a/b ratio may have increased owing to the photochemical oxidation of the light-harvesting complexes binding chlorophyll b (Huang et al., 2004).

As a rule, environmental stress caused by both biotic and abiotic factors leads to the formation and accumulation of reactive oxygen species in plant cells, which damage the cells and interfere with plant growth and yields. It is known that heavy metals can induce plant oxidative stress, the mechanisms and responses to which have been repeatedly described (Titov et al., 2014). In addition, the ability of bacteria to cause oxidative stress has been well documented (Rais et al., 2017). In response to oxidative explosion, various forms of plant antioxidants are activated, among which an important part is played by antioxidant defense enzymes. The activation of these enzymes under heavy metal stress has been described in detail (Titov et al., 2014). Rais et al. (2017) proposed that under stress caused by microbial infection, the antioxidant enzymes are activated in response to the recognition of microbial molecular patterns by the plant immune system; to some secondary metabolites of the microorganisms; and to plant iron status, altered by microbial siderophores. The changes in the activity of the antioxidant enzymes in wheat in response to various stress factors were summarized by Caverzan et al. (2016).

This study has shown how Azospirillum strains that use different strategies to colonize wheat roots in the presence of copper can affect the activity of peroxidase – an enzyme that, owing to its specific properties and a great variety its molecular forms, is a key protective cellular system that is used when any stress factors affect the plant (Statsenko et al., 2008). We have found that the activity of the peroxidase of wheat seedlings was significantly higher in roots than in shoots. The copper concentration used did not affect the activity of peroxidase in the roots and only slightly reduced it in the shoots of noninoculated plants. In turn, inoculation significantly changed peroxidase activity both in the absence and in the presence of copper. The inoculation effect was strain-specific. In plants grown without copper, peroxidase activity was significantly increased in roots when strains Sp245 and Cd were used and in shoots when strain Sp7 was used. By contrast, with copper, strain Sp7 strongly induced peroxidase activity in roots and to a lesser extent in shoots, whereas strains Sp245 and Cd promoted peroxidase activity mainly in shoots. Thus, inoculation caused a pronounced antioxidant stress response, in which various peroxidase isoforms were probably involved. The increase in copper uptake by the inoculated plants additionally promoted peroxidase activity, which shows a potentiated effect of the abiotic and biotic stress factors.

Besides peroxidases, phenol oxidases are almost universally present in plants. They are often induced under stress caused by damage to plants or by pathogen attack and are important for plant defense response (Sullivan, 2015). There is evidence (Yang et al., 2007) that biotic and abiotic stress activates plant lignin synthesis, which involves phenol oxidase and peroxidase. Our data show that the activity of these enzymes was affected by inoculation to a greater extent than it was affected by copper. Inoculation promoted an increase in the tissue concentration of copper, which, as a rule, promoted the activity of the phenol oxidases both as copper-dependent enzymes and as stress enzymes

The search for correlations between plant treatments and the variables analyzed showed a significant close correlation between the change in enzyme activity and the Azospirillum inoculation of plants (rs = 0.76, p < 0.05). It is noteworthy that among the strains tested, only the epiphyte A. brasilense Sp7 increased root enzyme activity in the presence of copper.

Thus, all the strains tested contributed to the uptake of copper by plants. We emphasize that the differences in the effects observed were caused by different strains. Thus, without copper, the endophytic strain A. baldaniorum Sp245 reduced the length but increased the weight of the seedling roots, contributed the least to the uptake of copper, and caused the least induction of the antioxidant enzymes and phenol oxidases. Inoculation of plants with the epiphytic strain A. brasilense Sp7 in the absence of copper increased shoot peroxidase and oxidase activity the most and contributed the most to the uptake of copper and to the activation of root peroxidases and oxidases in the presence of copper. The inbetween strain A. brasilense Cd promoted wheat growth the most, regardless of the presence of copper. Without copper, this strain increased root peroxidase and oxidase activity the most, and in the presence of copper, it inhibited the plant photosynthesis apparatus the most.

## Conclusion

Overall, the obtained results clearly show that the effect of Azospirillum on the physiological and biochemical status of wheat is diverse. The compensatory effect of bacteria on copper toxicity and the simultaneous increase in metal accumulation in plant tissues can be considered as mutually exclusive crop-production aspects associated with the growing of food plants in heavy-metal-polluted areas.

## Conflict of interest

The authors declare no conflict of interest.

## References

Bashan Y., de-Bashan L.E. How the plant growth-promoting bacterium
Azospirillum promotes plant growth – a critical assessment. Adv.
Agron. 2010;108:77-136. DOI 10.1016/S0065-2113(10)08002-8.

Boleta E.H.M., Shitate Galindo F., Jalal A., Santini J.M.K., Rodrigues
W.L., Lima B.H.D., Arf O., da Silva M.R., Buzetti S.,
Teixeira Filho M.C.M. Inoculation with growth-promoting bacteria
Azospirillum brasilense and its effects on productivity and nutritional
accumulation of wheat cultivars. Front. Sustain. Food Syst.
2020;4:607262. DOI 10.3389/fsufs.2020.607262.

Bradford M.M. A rapid and sensitive method for the quantitation of
microgram quantities of protein utilizing the principle of proteindye
binding. Anal. Biochem. 1976;72(1-2):248-254. DOI 10.1006/
abio.1976.9999.

Breckle S.W. Growth under stress: heavy metals. In: Waisel Y., Kafkafi
U. (Eds.) Plant Roots: The Hidden Half. N.Y.: Marsel Dekker
Inc., 1991;351-373.

Caiola M.G., Canini A., Botta A.L., Del Gallo M. Localization of Azospirillum
brasilense Cd in inoculated tomato (Lycopersicon esculentum
Mill.) roots. Ann. Microbiol. 2004;54(4):365-380.

Caverzan A., Casassola A., Brammer S.P. Antioxidant responses of
wheat plants under stress. Genet. Mol. Biol. 2016;39(1):1-6. DOI
10.1590/1678-4685-GMB-2015-0109.

Criquet S., Joner E., Leglize P., Leyval C. Anthracene and mycorrhiza
affect the activity of oxidoreductases in the roots and the rhizosphere
of lucerne (Medicago sativa L.). Biotechnol. Lett. 2000;22:1733-
1737. DOI 10.1023/A:1005604719909

Dang V.B.H., Doan H.D., Dang-Vu T., Lohi A. Equilibrium and kinetics
of biosorption of cadmium (II) and copper (II) ions by
wheat straw. Biores. Technol. 2009;100(1):211-219. DOI 10.1080/
19443994.2012.691745

de Oliveira Pinheiro R., Boddey L.H., James E.K., Sprent J., Boddey
R. Adsorption and anchoring of Azospirillum strains to roots
of wheat seedlings. Plant Soil. 2002;246(2):151-166. DOI 10.1023/
A:1020645203084

Dos Santos F.N., Hayashi Sant’ A.F., Massena R.V., Ambrosini A.,
Gazolla V.C., Rothballer M., Schwab S., Baura V.A., Balsanelli E.,
Pedrosa F.O., Pereira P.L.M., de Souza E.M., Hartmann A., Cassan
F., Zilli J.E. Genome-based reclassification of Azospirillum
brasilense Sp245 as the type strain of Azospirillum baldaniorum
sp. nov. Int. J. Syst. Evol. Microbiol. 2020;70:6203-6212. DOI
10.1099/ijsem.0.004517.

El-Samad A.H.M. The biphasic role of cupper and counteraction with
Azospirillum brasilense application on growth, metabolities, osmotic
pressure and mineral of wheat plant. Am. J. Plant Sci. 2017;8:
1182-1195. DOI 10.4236/AJPS.2017.85078.

Fukami J., Cerezini P., Hungria M. Azospirillum: benefits that go far
beyond
biological nitrogen fixation. AMB Express. 2018;8(1):73.
DOI 10.1186/s13568-018-0608-1.

Galindo F.S., Rodrigues W.L., Biagini A.L.C., Fernandes G.C., Baratella
E.B., da Silva C.A., Jr, Buzetti S., Teixeira Filho M.C.M. Assessing
forms of application of Azospirillum brasilense associated
with silicon use on wheat. Agronomy. 2019;9(11):678. DOI 10.3390/
agronomy9110678.

Hoagland D.R., Arnon D.I. The Water-Culture Method for Growing
Plants without Soil. Berkeley: Univ. of California, 1950;347:8.

Huang X.D., El-Alawi Y., Penrose D.M., Glick B.R., Greenberg B.M.
Responses of three grass species to creosote during phytoremediation.
Environ. Pollut. 2004;130(3):453-463. DOI 10.1016/j.envpol.
2003.12.018.

Kamnev A.A., Tugarova A.V., Antonyuk L.P. Endophytic and epiphytic
strains of Azospirillum brasilense respond differently to heavy
metal stress. Microbiology. 2007;76(6):809-811. DOI 10.1134/
S0026261707060239

Kamnev A.A., Tugarova A.V., Antonyuk L.P., Tarantilis P.A., Polissiou
M.G., Gardiner P.H.E. Effects of heavy metals on plant-associated
rhizobacteria: comparison of endophytic and non-endophytic
strains of Azospirillum brasilense. J. Trace Elem. Med. Biol. 2005;
19(1):91-95. DOI 10.1016/j.jtemb.2005.03.002.

Larionov М.V., Larionov N.V. Characteristics of accumulation of
heavy metals in soil ecosystems of Saratov Volga river region. Vestnik
Orenburgskogo Gosudarstvennogo Universiteta = Vestnik of the
Orenburg State University. 2010;1(107):110-114. (in Russian)

Leonowicz A., Grzywnowicz K. Quantitative estimation of laccase
forms in somewhite-rot fungi using syringaldazine as a substrate.
Enzyme Microb. Technol. 1981;3:55-58. DOI 10.1016/0141-0229
(81)90036-3.

Liu Y., Chen Y., Yang Y., Zhang Q., Fu B., Cai J., Guo W., Shi L., Wu J.,
Chen Y. A thorough screening based on QTLs controlling zinc and
copper accumulation in the grain of different wheat genotypes. Environ.
Sci. Pollut. Res. 2021;28:15043-15054. DOI 10.1007/s11356-
020-11690-3.

Lyubun Y., Muratova A., Dubrovskaya E., Sungurtseva I., Turkovskaya
O. Combined effects of cadmium and oil sludge on sorghum:
growth, physiology, and contaminant removal. Environ. Sci. Pollut.
Res. 2020;27:22720-22734. DOI 10.1007/s11356-020-08789-y.

Medvedev I.F., Derevyagin S.S. Heavy Metals in Ecosystems. Saratov:
Rakurs Publ., 2017. (in Russian)

Michaud A.M., Bravin M.N., Galleguillos M., Hinsinger P. Copper uptake
and phytotoxicity as assessed in situ for durum wheat (Triticum
turgidum durum L.) cultivated in Cu-contaminated, former vineyard
soils. Plant Soil. 2007;298(1-2):99-111. DOI 10.1007/s11104-007-
9343-0.

Nadeem S.M., Naveed M., Ahmad M., Zahir Z.A. Rhizosphere bacteria
for crop production and improvement of stress tolerance: mechanisms
of action, applications, and future prospects. In: Plant Microbes
Symbiosis: Applied Facets. India: Springer, 2015;1-36. DOI
10.1007/978-81-322-2068-8_1.

Pichhode M., Nikhil K. Effect of copper mining dust on the soil and
vegetation in India: a critical review. Int. J. Mod. Sci. Eng. Technol.
2015;2(2):73-76.

Prasad D.D.K., Prasad A.R.K. Altered 5-aminolevulinic acid metabolism
by lead and mercury in germinating seedlings of Bajra (Pennisetum
typhoideum). J. Plant Physiol. 1987;127:241-249. DOI
10.1007/BF02702668.

Prasad M.N.V., Strzalka K. Impact of heavy metals on photosynthesis.
In: Prasad M.N.V., Hagemeyer J. (Eds.) Heavy Metal Stress in
Plants: From Molecules to Ecosystems. Berlin: Springer, 1999;117-
138. DOI 10.1007/978-3-662-07745-0_6.

Quartacci M.F., Pinzino C., Sgherri C.L.M., Dalla Vecchia F., Navari-Izzo
F. Growth in excess copper induces changes in the lipid composition
and fluidity of PSII-enriched membranes in wheat. Physiol. Plantarum.
2000;108:87-93. DOI 10.1034/j.1399-3054.2000.108001087.x.

Rai R., Agrawal M., Agrawal S.B. Impact of heavy metals on physiological
processes of plants: with special reference to photosynthetic
system. Ch. 6. In: Singh A., Prasad S.M., Singh R.P. (Eds.) Plant
Responses to Xenobiotics. Singapore: Springer Nature, 2016;127-
140. DOI 10.1007/978-981-10-2860-1_6

Rais A., Jabeen Z., Shair F., Hafeez F.Y., Hassan M.N. Bacillus spp.,
a bio-control agent enhances the activity of antioxidant defense enzymes
in rice against Pyricularia oryzae. PLoS One. 2017;12(11):
e0187412. DOI 10.1371/journal.pone.0187412.

Reis V., Baldani V.L.D., Baldani J.I. Isolation, identification and biochemical
characterization of Azospirillum spp. and other nitrogenfixing
bacteria. In: Cassán F., Okon Y., Creus C. (Eds.) Handbook for
Azospirillum. Basel: Springer, 2015;10:978-983. DOI 10.1007/978-
3-319-06542-7_1.

Rothballer M., Schmid M., Hartmann A. In situ localization and PGPReffect
of Azospirillum brasilense strains colonizing roots of different
wheat varieties. Symbiosis. 2003;34:261-279.

Sayyad G., Afyuni M., Mousavi S.-F., Abbaspour K.C., Hajabbasi
M.A., Richards B.K., Schulin R. Effects of cadmium, copper,
lead, and zinc contamination on metal accumulation by safflower
and wheat. Soil Sediment Contam. Int. J. 2009;18(2):216-228. DOI
10.1080/15320380802660248.

Statsenko A.P., Tuzhilova L.I., Vyugovsky A.A. Plant peroxidases
– markers of chemical pollution of natural environments. Vestnik
Orenburgskogo Gosudarstvennogo Universiteta = Vestnik of the
Orenburg State University. 2008;10(92):188-191. (in Russian)

Sullivan M.L. Beyond brown: polyphenol oxidases as enzymes of plant
specialized metabolism. Front. Plant Sci. 2015;5:783. DOI 10.3389/
fpls.2014.00783.

Teixeira Filho M.C.M.T., Galindo F.S., Buzetti S., Santini J.M.K. Inoculation
with Azospirillum brasilense improves nutrition and increases
wheat yield in association with nitrogen fertilization. Ch. 6. In:
Wanyera R., Owuoche J. (Eds.) Wheat Improvement, Management
and Utilization. IntechOpen, 2017;99-114. DOI 10.5772/67638.

Titov A.F., Kaznina N.M., Talanova V.V. Heavy Metals and Plants.
Petrozavodsk: Karelian Research Centre of RAS, 2014. (in Russian)

Wang H., Zhong G., Shi G., Pan F. Toxicity of Cu, Pb, and Zn on seed
germination and young seedlings of wheat (Triticum aestivum L.).
In: Li D., Liu Y., Chen Y. (Eds.) Computer and Computing Technologies
in Agriculture IV. CCTA 2010. IFIP Advances in Information
and Communication Technology. Vol. 346. Berlin; Heidelberg:
Springer, 2011;231-240. DOI 10.1007/978-3-642-18354-6_29.

Wang S., Wu W., Liu F., Liao R., Hu Y. Accumulation of heavy metals
in soil-crop systems: a review for wheat and corn. Environ. Sci.
Pollut. Res. 2017;24(18):15209-15225. DOI 10.1007/s11356-017-
8909-5.

Yaneva O.D. Mechanisms of bacterial resistance to heavy metal
ions.
Mikrobiologichnyi Zhurnal = Microbiological Journal. 2009;
71(6):54-65. (in Ukrainian)

Yang Y.-J., Cheng L.-M., Liu Z.-H. Rapid effect of cadmium on lignin
biosynthesis in soybean roots. Plant Sci. 2007;172:632-639. DOI
10.1016/j.plantsci.2006.11.018.

Yruela I. Cooper in plants. Braz. J. Plant Physiol. 2005;17(1):145-156.
DOI 10.1590/S1677-04202005000100012.

